# Mechanism of Action of Botulinum Toxin A in Treatment of Functional Urological Disorders

**DOI:** 10.3390/toxins12020129

**Published:** 2020-02-18

**Authors:** Yu-Hua Lin, Bing-Juin Chiang, Chun-Hou Liao

**Affiliations:** 1 Divisions of Urology, Department of Surgery, Cardinal Tien Hospital, New Taipei City 23148, Taiwan; jorgesuperowl@gmail.com (Y.-H.L.); bingjuinchiang@gmail.com (B.-J.C.); 2 Department of Chemistry, Fu Jen Catholic University, New Taipei City 24205, Taiwan; 3 Graduate Institute of Biochemical and Pharmaceutical Science, Fu Jen Catholic University, New Taipei City 24205, Taiwan; 4 College of Medicine, Fu-Jen Catholic University, New Taipei City 24205, Taiwan; 5 School of Life Science, College of Science, National Taiwan Normal University, Taipei 11677, Taiwan

**Keywords:** botulinum toxin, functional urology disorder, human

## Abstract

Intravesical botulinum toxin (BoNT) injection is effective in reducing urgency and urinary incontinence. It temporarily inhibits the detrusor muscle contraction by blocking the release of acetylcholine (Ach) from the preganglionic and postganglionic nerves in the efferent nerves. BoNT-A also blocks ATP release from purinergic efferent nerves in the detrusor muscle. In afferent nerves, BoNT-A injection markedly reduces the urothelial ATP release and increases nitric oxide (NO) release from the urothelium. BoNT-A injection in the urethra or bladder has been developed in the past few decades as the treatment method for detrusor sphincter dyssyndergia, incontinence due to neurogenic or idiopathic detrusor overactivity, sensory disorders, including bladder hypersensitivity, overactive bladder, and interstitial cystitis/chronic pelvic pain syndrome. Although the FDA only approved BoNT-A injection treatment for neurogenic detrusor overactivity and for refractory overactive bladder, emerging clinical trials have demonstrated the benefits of BoNT-A treatment in functional urological disorders. Cautious selection of patients and urodynamic evaluation for confirmation of diagnosis are crucial to maximize the successful outcomes of BoNT-A treatment.

## 1. Introduction

Botulinum toxin (BoNT), one of the most potent natural neurotoxins known for centuries, has been found with emerging medical efficacy in the past few decades [1,2]. BoNT was initially documented with the symptoms of foodborne botulism in the 18th century [3]. A botulism outbreak after a funeral dinner with smoked ham in 1895 led to the discovery of the pathogen Clostridium botulinum by Emile Pierre van Ermengem, Professor of Bacteriology at the University of Ghent [3]. Acute BoNT poisoning was initially observed with vomiting, intestinal spasms, mydriasis, ptosis, dysphagia, and finally respiratory failure [4]. It may take 3–6 months to recover from botulinum intoxication [4]. Since BoNT was discovered as the produced toxin from the bacterium Clostridium botulinum, it has been widely used to treat neuropathic pain syndromes and dystonic disease [5,6,7,8].

Botulinum toxin A (BoNT-A) has been used for the treatment of lower urinary tract disease (LUTD) since the late 1980s. Dykstra et al. reported injection of BoNT-A to the external urethral sphincter in men with spinal cord injury (SCI) for the treatment of detrusor-sphincter dyssynergia (DSD) in 1988 [9]. The treatment of SCI patients with neurogenic detrusor overactivity (DO) using detrusor BoNT-A injections at multiple sites was also developed [10]. Idiopathic DO and overactive bladder (OAB) patients were also reported with successful treatment with intravesical BoNT-A injection [11,12]. Maria et al. first described the therapeutic effects of BoNT-A injection for patients with benign prostatic hyperplasia (BPH) with voiding dysfunction in 2003 [13]. However, the latest randomized controlled trial investigating the efficacy of BoNT-A injection for BPH-related lower urinary tract symptoms (LUTS) demonstrated no significant difference between the treatment group and the placebo [14]. Moreover, BoNT-A intravesical injection treatment has been developed for interstitial cystitis/bladder pain syndrome (IC/BPS) because of its anti-inflammatory effects [15,16]. As the uses of BoNT-A expand in the field of urology, understanding its mechanisms and clinical effects is essential.

## 2. Mechanism of Action of BoNT-A

BoNT is a neurotoxin protein, which comprises a 50-kDa light chain and a 100 kDa heavy chain linked by a disulfide bond [17]. Seven serotypes of BoNT has been identified, and the most commonly used type in medicine is BoNT-A [17]. BoNT enters the presynaptic neuron membrane through binding of the heavy-chain C-terminal to the synaptic vesicle protein (SV2) [18]. After toxin endocytosis, the disulfide bond of BoNT is cleaved. The light-chain protein, which is the true active moiety, is then linked to the synaptosomal nerve associated protein 25 (SNAP-25) [18]. SNAP-25 is a protein with essential function for the binding of vesicles to the cell membrane and signal transduction. By binding the light-chain protein of BoNT-A to SNAP-25 and other SNAP families, BoNT-A inhibits neurotransmitters’ exocytosis from the vesicles; hence, the affected neuromuscular junctions become paralyzed [18].

A clinical study confirmed SV2 and SNAP-25 immunoreactive fibers are distributed over the suburothelial and muscular layers instead of the urothelium in human bladder [19]. SV2 or SNAP-25 protein is not expressed within the urothelial or muscular cells [19]. The SV2 are expressed more abundantly in the cholinergic and parasympathetic fibers, as compared to the less than half expression to the sensory and sympathetic nerves. These findings suggest that the parasympathetic nerves are the main target of BoNT-A action in the human urinary bladder [19]. Other clinical studies associated with animal models demonstrated the SV2 expression in the human and rat bladder mucosae, as well as synaptosomal nerve-associated protein 23 (SNAP-23) and SNAP-25 in the urothelial cells and mucosa (differed in intensity) from the rat and human bladder [20]. SNAP-23 is a homologous target membrane SNAP receptor (t-SNARE) and is structurally and functionally similar to SNAP-25. SNAP-23 may be cleaved by BoNT-A, but human SNAP-23 is more resistant to botulinum [21,22]. The distribution pattern of SNAP-23 is different from that of SNAP-25: SNAP-23 is expressed mainly within the superficial or apical layer of urothelial layer, while SNAP-25 is detected throughout the urothelial layer [20]. SNAP-23 also interacts to multiple vesicle-associated membrane protein and syntaxin [23]. Since the urothelium is considered both a barrier as well as a significant signal transduction gate, the release of other neurotransmitters such as glutamate, adenosine triphosphate (ATP), neurotrophins, or NO may be affected after BoNT-A injection [24,25].

In clinical studies, BoNT-A inhibits the release of acetylcholine (Ach) and other neurotransmitters at the neuromuscular junction in human striated muscle [26]. Further neural modulating effects are observed by influencing the α and γ motor neurons after BoNT-A treatment (the α and γ motor neurons innervate the extrafusal and the intrafusal muscle fibers, respectively) [26]. Intravesical BoNT-A administration results in SNAP-25 cleavage, which inhibits the vesicular noradrenaline release. This action may prevent the α- and β3-adrenoceptors activation, and the reaction additionally affects the bladder neck contracture and detrusor relaxation [27]. A clinical study with receptor analysis conducted after BoNT-A injection treatment to the human bladders with neurogenic detrusor overactivity (NDO) showed significant reduction of the M2 and M3 muscarinic receptors as well as the P2X purinoceptor 2 (P2X2) and P2X purinoceptor 3 (P2X3) in the muscle layer [28]. This indicates that BoNT-A hinders DO through both sensory and motor features.

In addition, the ATP receptor P2X3 is critical for peripheral pain responses and afferent pathways controlling the urinary bladder volume reflexes [29,30,31]. In an animal model study, P2X3-null mice presented a marked hyporeflexia of the urinary bladder [29]. This result indicates that ATP plays an important role in mediating bladder fullness sensation and is crucial in the pathophysiology of OAB. In clinical studies, the human bladder, P2X3, and the transient receptor potential vanilloid subfamily-1 (TRPV1) are observed in the suburothelial layer [30]. A clinical study on the receptor profiles in biopsies from NDO or idiopathic DO patients showed a decreased P2X3 and TRPV1 immunoreactivity in the sensory nerve fiber after BoNT-A intravesical injection treatment [31]. The degree of decrease in the TRPV1 and P2X3 immunoreactivity is found to be correlated to clinical improvement (reduction of frequency and urgency status) [31]. Clinical studies with intravesical BoNT-A injections demonstrated significant inhibition of ATP and neurotrophin release and an increase of nitric oxide (NO) release from the human urothelial cells [24,30].

Animal models have shown possible mechanisms of action of BoNT-A injection treatment in interstitial cystitis/bladder pain syndrome (IC/BPS). In the isolated rat bladder model of acute injury and chronic inflammation, a significant amount of reduced calcitonin gene-related peptide and substance P from the afferent nerve terminals is observed [32,33]. The results suggest that BoNT-A injection treatment is a solution method of neurogenic inflammation in patients with IC/BPS [32,33]. TRPV1 inflammatory sensitization is found to play a vital role in inflammatory pain mediation [34]. Some proinflammatory agents (e.g., nerve growth factor, ATP, and IGF-I) sensitize rat nociceptors by promoting the recruitment of TRPV1 channels to the neuronal surface [34]. In preclinical studies, BoNT-A injection into oocytes expressing TRPV1 was found to block the TRPV1 membrane translocation by affecting protein kinase C (PKC) signaling [35]. The inhibition of the inflammatory sensitization of TRPV1 receptors by BoNT-A may also describe the therapeutic effects of BoNT-A injection to medication refractory IC/BPS. In an animal model study, BoNT-A has been shown to inhibit the ATP release from the urothelium in chronic bladder inflammation [36]. In clinical studies, reduction of the nerve growth factor and brain-derived neurotrophic levels in patients with IC/BPS after intravesical BoNT-A injection has demonstrated an analgesic effect [37,38]. BoNT-A conducted direct analgesic effects through exocytosis suppression of sensory neurotransmitters over the peripheral nociceptive neurons. However, indirect analgesic effects seemed also present with decreased spinal cord neuronal activity and with prevention of central sensitization as verified in some other clinical studies [39,40].

In a preclinical study for LUTS that related to prostate enlargement, BoNT-A injection has been reported to induce prostate atrophy and activate the apoptotic pathway in rats, which may result in reduced sympathetic stimulation of the prostate [41]. BoNT-A injection in a rat model revealed prostate weight reduction and reduced level of tyrosine hydroxylase-positive sympathetic nerve fibers and synaptophysin-positive cells in the epithelium [42,43]. In clinical studies, human prostate injection of BoNT-A has demonstrated apoptotic activity at the epithelial and stromal components of the prostate [44]. The reaction subsequently reduced the anatomical obstruction [44]. A clinical study comparing intraprostatic BoNT-A and normal saline injections demonstrated a significant contractile function reduction while maintaining relaxation response by presenting reduced prostatic urethral pressure response to intravenous norepinephrine and electrostimulation [45].

## 3. BoNT-A Treatment in OAB and DO

OAB is a clinical syndrome characterized by urinary urgency, usually accompanied by frequency and nocturia, with or without urinary incontinence, in the absence of urinary tract infection or other pathology [46]. Treatment of OAB is typically initiated with behavioral therapy, followed by oral medications including antimuscarinics or beta-agonists [47]. A large-scale study showed 46.2% of OAB patients discontinued medical treatment due to poor response or less effective as expected after treatment [48]. DO was defined as a urodynamic observation characterized by involuntary detrusor contraction during the bladder-filling phase [49]. DO is usually associated with symptoms of urgency, which is defined as a complaint of sudden, compelling desire to pass urine that is difficult to defer [50]. DO has been noticed in those with disturbances in the nerve, detrusor muscular, or urothelial levels [50]. Ach plays a key role in mediating bladder contraction through muscarinic receptors and detrusor muscle, and mediating ATP through purinergic receptors (P2X) stimulation has also been associated with bladder contraction [30].

In the central nervous system (CNS), the prefrontal cerebral cortex, the L-region of the pontine micturition center, and the lumbar spinal cord play the essential role of detrusor contraction inhibition [50]. A spinal cord lesion above the lumbosacral cord level may cause inhibitory pathway dysfunction, which further disturbs the voluntary control of micturition and results in NDO [50]. The sacral spinal reflex is known to be mediated by the unmyelinated C-fibers and is active in patients with SCI and NDO [50]. For idiopathic detrusor overactivity (IDO), although there is no specific central or peripheral neurologic dysfunction, increased expression of P2X2, TRPV1, and muscarinic receptors over the urothelium have been found in a clinical study [51].

Several clinical studies have demonstrated BoNT-A’s efficacy in urgency and urinary incontinence reduction [10]. In the efferent nerves, BoNT-A injection temporarily inhibits the detrusor muscle contraction by blocking the Ach release via cleaving SNAP-25 from both the preganglionic and postganglionic nerve [24]. BoNT-A also blocks ATP release from the purinergic efferent nerves in the detrusor muscle [52]. In the afferent nerves, BoNT-A injection markedly reduced urothelial ATP release [53,54]. NO inhibits the afferent nerve conduction in the bladder detrusor and BoNT-A injection facilitates increased NO release from the urothelium [54]. In summary, BoNT-A injection has effects involving the efferent and afferent nerve pathways.

## 4. BoNT-A Treatment for DSD in Patients with Spinal Cord Injury

DSD is characterized by involuntary contraction of the external urethral sphincter during a detrusor contraction and is caused by CNS injury between the sacral spinal cord and pontine micturition center [55]. High post-void urine amount, incomplete emptying, and increased intravesical pressure during voiding phase are noticed in the SCI patient with DSD. BoNT-A injection to the urethral sphincter has demonstrated sphincter relaxation by blocking Ach from the presynaptic vesicles at the neuromuscular junction [9,56,57]. Although DSD patient receiving BoNT-A injection over the urethral sphincter may experience incontinence, most of the patients were satisfied by the treatment effects of significant urethral pressure reduction, increased voiding volume, and decreased urinary incontinence episodes [56,57].

## 5. BoNT-A Injection for Dysfunctional Voiding (DV) or Bladder Neck Dysfunction (BND)

DV is characterized by voiding intermittency or urine flow rate fluctuation due to involuntary contraction of the periurethral striated muscle during voiding in non-neurological deficit individuals [49]. As BoNT-A injection treatment has been successfully used in SCI patients with DSD, it has been developed for adults with non-neurogenic voiding dysfunction. Fowler’s syndrome consists of challenging symptoms of poor external urethral sphincter relaxation without neurological or anatomical abnormality, which may cause voiding difficulty and even urinary retention [58]. Patients with non-neurogenic voiding dysfunction classically present with open bladder neck but poorly relaxed urethral sphincter and normal-to-high voiding pressure during micturition by urodynamic study and are mostly refractory to medical treatment [59]. Several recent clinical studies have shown BoNT-A injection to the external urethral sphincter could be a safe and efficient treatment method for refractory DV patients [60,61,62]. Post-treatment, improvements were noticed in several clinical aspects in those studies, such as maximal flow rate, voided urine amount, decreased detrusor voiding pressure, international prostate symptom score (IPSS), and quality of life index. However, only the total IPSS and voided volume improvements were significantly greater than those in the placebo group in a randomized controlled double-blind study [62].

Some DV patients without strong electromyographic activity may have similar symptoms of DV due to poor relaxation of the pelvic floor muscle and the urethral sphincter [63]. Poor bladder neck relaxation in BND may lead to weak stream and increased residual urine amount after voiding [63]. Recent studies demonstrated that BoNT-A injections in the external urethral sphincter, pelvic floor muscle, or bladder neck may offer promising therapeutic effects for DV symptoms improvement [61,64,65]. Synchronous significant reduction of the bladder outlet resistance and the pelvic floor pressure were observed in a study after BoNT-A injection treatment to the pelvic floor muscle, which indicates a more complex mechanism in DV symptoms. Further evidence is necessary to explain the pathophysiology of BoNT-A action in DV or bladder neck dysfunction.

## 6. BoNT-A Injection for IC/BPS

IC/BPS is a clinical syndrome described as having “An unpleasant sensation (pain, pressure, discomfort) perceived to be related to urinary bladder, associated with lower urinary tract symptoms of more than 6 weeks duration, in the absence of infection or other identifiable causes” [62]. Though the real pathophysiology of IC/BPS has remained unclear for decades, recent studies have progressed in molecular biology, which have focused on urothelial dysfunction and neurogenic inflammation and could explain some part of the disease [61,62,63,64,65,66]. Urothelial defect with surface glycosaminoglycan and associated dysregulation of urothelial permeability has been established as one of the pathogenesis of IC/BPS [65]. IC/BPS also had upregulated P2X3 receptors and with increasing ATP release in the urothelium [66]. TRPV1 is a capsaicin receptor that detects bladder pain. A recent clinical study found that the increased inflammation severity correlated with a high TRPV1-immunoreactive nerve fibers and nerve growth factor (NGF) in IC/BPS expression and the correlation is directly positive to the clinical symptoms [67]. Substance P, a neurotransmitter secreted from the sensory nerve and a key cytokine in inflammatory process and pain, was also found with increased expression in the bladder nerve fibers of IC/BPS patients [68]. NGF, a neuropeptide involved in the regulation of growth released by the mast cell in the inflammatory process, was found with increased expression in the bladder mucosa, urine, and serum of IC/BPS patients [69,70]. NGF is also believed to play a pivotal role in the pathogenesis of IC/BPS.

BoNT-A injections improved urothelial function in IC/BPS patients by P2X3 and TRPV1 receptor expression reduction in the urothelium, which may be the chronic pain control mechanism [71]. BoNT-A injections inhibit the sensory neurotransmitters that further reduce pain sensation [71]. Various clinical studies have shown decreased NGF mRNA expression in the bladder for those who responded to intravesical BoNT-A injection treatment [72,73]. Recent clinical evidence suggests that BoNT-A stops the inflammation process in the bladder [74,75]. Declined tryptase expression was discovered over the urothelium after repeated BoNT-A injection, which indicated the reduction of mast cell activity in the treated IC/BPS patients’ bladder [74]. Furthermore, BoNT-A injection treatment was found with decreased vascular endothelial growth factor (VEGF) and attenuated vasculogenesis in the bladder of IC/BPS patients [75]. Apoptotic signaling also decreased in evidence after BoNT-A injection treatment [75].

## 7. BoNT-A Injection for BPH

BPH is the most common cause of bladder outlet obstruction in men. The prostate growth’s anatomical obstruction may be the main reason for lower urinary tract symptoms, but inflammation, infection, and metabolic disorders can also be possible etiologies [76]. The prostate is innervated with the parasympathetic and sympathetic nerves [77]. The cholinergic nerves and muscarinic receptors are present in the fibromuscular stroma of the prostate, which can explain the previous beneficial effects of BoNT-A intraprostatic injection treatments [78,79,80,81]. However, a recent randomized controlled trial stated that high placebo effects appeared persistent and may be associated with the symptom improvement [14]. Several studies have shown that BoNT-A injection into prostate can effectively reduce pain in men with chronic prostatitis [82]. The effect of BoNT-A on male LUTS might result from anti-inflammation effect but not reducing the prostate volume [82]. Currently, BoNT-A injection treatment remains the less-common indication for men with BPH and lower urinary tract symptoms.

## 8. Conclusions

BoNT-A injections are widely used in functional urology disorders, and therapeutic benefits have been noticed across a wide range of lower urinary tract diseases. However, approval for indication has been obtained only for management of idiopathic OAB and NDO owing to SCI or multiple sclerosis. The United States and some other countries also approved use for the detrusor hyperactivity with impaired contractile function. Moreover, for IC/BPS patients’ refractory to medical treatment, BoNT-A injections are considered the standard of treatment according to different guidelines. The pathophysiology of BoNT-A injection to different functional disorders is increasingly revealed in various clinical trials, and with promising therapeutic results of novel BoNT-A applications, including DV, BPH, and chronic prostatitis. However, not all patients of functional urology disorders refractory to conventional therapy can benefit from BoNT-A treatment, possibly because the underlying pathophysiology of these diseases has not been entirely clarified.

## List of Abbreviations

Ach	acetylcholine
ATP	adenosine triphosphate
BND	bladder neck dysfunction
BoNT	botulinum toxin
BoNT-A	Botulinum toxin A
BPH	benign prostatic hyperplasia
CNS	central nervous system
DO	detrusor overactivity
DSD	detrusor-sphincter dyssynergia
DV	dysfunctional voiding
IC/BPS	interstitial cystitis/bladder pain syndrome
IDO	idiopathic detrusor overactivity
IPSS	international prostate symptom score
LUTD	lower urinary tract disease
LUTS	lower urinary tract symptoms
NDO	neurogenic detrusor overactivity
NGF	nerve growth factor
NO	nitric oxide
OAB	overactive bladder
P2X	purinergic receptors
P2X2	P2X purinoceptor 2
P2X3	P2X purinoceptor 3
PKC	protein kinase C
SCI	spinal cord injury
SNAP	synaptosomal nerve associated protein
SNAP-25	synaptosomal nerve associated protein 25
SNAP-23	synaptosomal nerve associated protein 23
SNARE	SNAP receptor
SV2	synaptic vesicle protein
t-SNARE	target membrane SNAP receptor
TRPV1	transient receptor potential vanilloid subfamily-1
VEGF	vascular endothelial growth factor
